# Influence of Barotropic Tidal Currents on Transport and Accumulation of Floating Microplastics in the Global Open Ocean

**DOI:** 10.1029/2019JC015583

**Published:** 2020-01-29

**Authors:** Miriam F. Sterl, Philippe Delandmeter, Erik van Sebille

**Affiliations:** ^1^ Institute for Marine and Atmospheric Research Utrecht University Utrecht The Netherlands

**Keywords:** marine plastic, tidal currents, surface transport, Lagrangian modeling

## Abstract

Floating plastic debris is an increasing source of pollution in the world's oceans. Numerical simulations using models of ocean currents give insight into the transport and distribution of microplastics in the oceans, but most simulations do not account for the oscillating flow caused by global barotropic tides. Here, we investigate the influence of barotropic tidal currents on the transport and accumulation of floating microplastics, by numerically simulating the advection of virtual plastic particles released all over the world's oceans and tracking these for 13 years. We use geostrophic and surface Ekman currents from GlobCurrent and the currents caused by the four main tidal constituents (M ${}_{2}$, S ${}_{2}$, K ${}_{1}$, and O ${}_{1}$) from the FES model. We analyze the differences between the simulations with and without the barotropic tidal currents included, focusing on the open ocean. In each of the simulations, we see that microplastic accumulates in regions in the subtropical gyres, which is in agreement with observations. The formation and location of these accumulation regions remain unaffected by the barotropic tidal currents. However, there are a number of coastal regions where we see differences when the barotropic tidal currents are included. Due to uncertainties of the model in coastal regions, further investigation is required in order to draw conclusions in these areas. Our results suggest that, in the global open ocean, barotropic tidal currents have little impact on the transport and accumulation of floating microplastic and can thus be neglected in simulations aimed at studying microplastic transport in the open ocean.

## Key Points

- Barotropic tidal currents have little impact on transport and accumulation of floating microplastic in the global open ocean
- Simulations of plastic dispersion in the open ocean do not need to take barotropic tides into account
- A computationally efficient implementation for advecting virtual particles by tidal currents is provided

## 1 Introduction

Marine pollution caused by floating plastic debris is a global problem of increasing concern (e.g., Andrady, 2011; Law, 2016). Plastic litter can enter the oceans from land‐based sources or entry points including rivers (Lebreton et al., 2017), beaches, and agricultural runoff or sea‐based sources such as ships, platforms, and fishing piers (see Maximenko et al., 2019, for a recent review) and is now one of the most common and persistent pollutants in the global ocean. Plastic waste floating at the ocean surface can significantly harm the near‐surface ocean environment, especially marine life (e.g., Compa et al., 2019).

Understanding the severity of the harm marine plastic litter can cause, and finding solutions to the growing problem of plastic waste accumulating in the oceans, requires knowledge of the sources, pathways, and fate of plastics. However, in most of the world's oceans, data on plastic debris are very scarce (van Sebille et al., 2015). Sampling microplastics is especially hard as they are not easily observable due to their small size (Maximenko et al., 2019). It is therefore very challenging to study microplastic abundance, distributions, and pathways purely from observational data. Instead, simulations using numerical models of ocean currents can help us improve our understanding of the transport and fate of microplastics in marine environments (Hardesty et al., 2017). Many of these simulations use models forced by wind and buoyancy (e.g., Cózar et al., 2014; Jalón‐Rojas et al., 2019; Iwasaki et al., 2017; Lacerda et al., 2019; Lebreton et al., 2012; Wichmann et al., 2019).

Tidal forcing is often not included in the context of microplastic transport. The barotropic currents caused by tidal forces are typically 1 or 2 orders smaller in magnitude than the wind‐ and buoyancy‐driven surface currents in the open ocean and are therefore often expected to have a small influence on the transport of microplastic there. Nonetheless, barotropic tidal currents are in general leading order in coastal areas, which are particularly relevant for plastic transport and accumulation (Zhang, 2017) as most plastic enters the ocean in coastal environments (Cole et al., 2011). Furthermore, many of the plastics released into the ocean stay near the coastline for a long time (Lebreton et al., 2012), and high relative concentrations of microplastics have been observed in near‐shore areas (e.g., Auta et al., 2017; Desforges et al., 2014). It is unclear if the tides also have an effect on microplastic transport in the open ocean. Studying the influence of barotropic tidal currents on the transport of microplastics can be useful to help us understand the transport and distribution of microplastics in oceans. Moreover, it can help us determine the relevance of including barotropic tidal currents in simulations used to investigate the global transport of microplastics.

Here, we investigate the impact of barotropic tidal currents on the transport and distribution of microplastics in the global open ocean on time scales of months to years. We do this by simulating the advection of microplastic particles by ocean surface currents and comparing the outcomes with and without barotropic tidal currents included. Note that our study does not consider baroclinic tides or internal waves at tidal frequencies. Henceforth, unless explicitly stated otherwise, “tides” and “tidal currents” will refer to barotropic tides and barotropic tidal currents.

## 2 Methods

### 2.1 Harmonic Analysis of the Tides

Variations in the relative positions and orientations of the Earth, Moon, and Sun cause temporal variations in the tides. These astronomical cycles produce a great number of tidal constituents contributing to the tides, each of which has a period corresponding with the period of one of these cycles. The global tide can be decomposed into these tidal constituents by using harmonic analysis. The equations for the horizontal tidal velocity currents U and V at any time t and at any location $\vec{x}$ are given by the following expressions (Godin, 1972; Schureman, 1958):

(1) $$\begin{array}{ccc} U\left(\vec{x},t\right) & =\underset{i}{\sum }f_{i}(t)A_{i}^{U}(\vec{x})\cos\left[\omega_{i}(t-t_{0})+u_{i}(t)+V_{i}(t_{0})-\varphi_{i}^{U}\left(\vec{x}\right)\right], & \\ V\left(\vec{x},t\right) & =\underset{i}{\sum }f_{i}(t)A_{i}^{V}(\vec{x})\cos\left[\omega_{i}(t-t_{0})+u_{i}(t)+V_{i}(t_{0})-\varphi_{i}^{V}\left(\vec{x}\right)\right], \end{array}$$

where U and V denote the zonal and meridional components of the velocity caused by the tidal forces, respectively. The location on Earth (latitude and longitude) is denoted by the vector $\vec{x}$. The variable t represents the time, measured with respect to some initial time $t_{0}$. The index i denotes the different tidal constituents, all of which have their own angular frequency $\omega_{i}$. The coefficients $A_{i}^{U}$ and $A_{i}^{V}$ are the amplitudes of constituent i; $\varphi_{i}^{U}$ and $\varphi_{i}^{V}$ are the initial phases or phase shifts of constituent i at time $t_{0}$. These amplitudes and phase shifts depend on longitude and latitude and can be determined from observational data. Furthermore, there are a number of time‐dependent correction factors. The astronomical argument correction $V_{i}(t_{0})$ reexpresses the phase shifts with respect to an absolute time origin ( $t_{0}$) (Foreman & Henry, 1989). The variables $f_{i}(t)$ and $u_{i}(t)$ are the nodal modulation amplitude and phase corrections, respectively, and account for the slow time evolution of the amplitude and phase of a constituent (Foreman, 1977; Foreman & Henry, 1989).

Here, we will use only the four largest tidal constituents (M ${}_{2}$, S ${}_{2}$, K ${}_{1}$, and O ${}_{1}$) and will refer to these four constituents as the main tidal constituents and to the currents they cause as the main tidal currents. See Text S1 in the supporting information for a more elaborate discussion on these components.

### 2.2 Data Set for Tidal Currents

For the tidal currents, we use the FES2014 data set (Carrère et al., 2015), which provides the amplitudes and phase shifts of the eastward and northward velocity component of 34 tidal constituents on a 1/16° resolution grid. As mentioned above, here only the M ${}_{2}$, S ${}_{2}$, K ${}_{1}$, and O ${}_{1}$ constituents are taken into account. Note that the amplitudes and phase shifts from these data depend only on latitude and longitude (see equation (1)). Maps of the tidal amplitudes of these four constituents can be found in Figures S1 and S2 in the supporting information. The full space‐ and time‐dependent main tidal currents are calculated as discussed in Text S1 in the supporting information.

### 2.3 Data Set for Ocean Surface Currents

We use the GlobCurrent v3 data set for ocean surface currents (total_hs) for the period of 2002–2014. This data set contains values of the eastward and northward velocity components at the ocean surface on a global grid with spatial resolution 1/4° and with a temporal resolution of 1 day and was recently used by Onink et al. (2019) to study the separate effects of the geostrophic, Ekman and Stokes drift flow to floating microplastic accumulation.

The GlobCurrent project combines satellite measurements with in situ measurements to obtain estimates of ocean surface currents (Rio et al., 2014, 2015), based on altimetry data, ARGO float drifts at the ocean surface, and undrogued surface drifters from the Global Drifter Program (e.g., Lumpkin et al., 2017). The total surface currents in the GlobCurrent data set are the sum of the geostrophic velocities and the surface Ekman velocities. A time‐averaged map of these total currents can be found in Figure S3 in the supporting information.

The advantage of using GlobCurrent and FES over a fine‐resolution Ocean General Circulation Model simulation with tides (e.g., Torres et al., 2018) is that the GlobCurrent and FES data are strongly constrained by observations and that the simulations can extend much longer, facilitating analysis on time scales relevant to the accumulation of plastic into the centers of the ocean gyres.

### 2.4 Particle Tracking Simulations

For our numerical simulations, we use Parcels (Probably A Really Efficient Lagrangian Simulator) v1.1 (Lange & van Sebille, 2017) to model microplastics as virtual particles which are advected using ocean flow field data. Parcels computes Lagrangian particle trajectories (e.g., van Sebille et al., 2018) by integrating the linearly interpolated velocity field data. To model particle advection by tidal currents, we developed a Kernel that for every time step computes the zonal and meridional velocities caused by the main tidal constituents at that time at the particle location (as described in Text S1 in the supporting information), using the data from FES2014 for the amplitudes and phase shifts. The code used for this can be found on GitHub (https://github.com/OceanParcels/Tides_GlobalOceanPlastic).

In this project, the velocity fields used to integrate the particles are the sum of the geostrophic and Ekman velocities and the tidal current velocities. This means that we do not take into account any nonlinear interactions between the tidal flow and the GlobCurrent flow, which is a reasonable assumption in the open ocean away from coasts because the spatial scale of the barotropic tides is so much larger than that of the GlobCurrent flow. Another important note is that all the currents used here are two dimensional; the model does not account for vertical motion such as sinking, and thus, all the particles in the simulation will stay on the ocean surface.

In our simulations, we release an initial homogeneous microplastic distribution, with particles placed in oceans at 1° intervals for latitudes between 75°S and 75°N and longitudes between 179.75°W and 179.75°E (34,370 particles in total). We have also done a simulation with four times as many particles, and results are similar. To integrate the particle trajectories, we use the classical fourth‐order Runge‐Kutta method. To investigate the influence of tides on the transport and trajectories of microplastic, we perform three different simulations: one where the particles are advected only by the geostrophic and Ekman currents, one where they are advected only by the main tidal currents, and one with all these currents combined. For simplicity, these simulations will henceforth be referred to as the GC (GlobCurrent) simulation, the FES simulation, and the GC+FES simulation, respectively.

A fourth simulation is done with the same conditions as the GC+FES simulation, but now the advection by barotropic tidal currents is included only for the first 30 days, and afterward only the GlobCurrent data are used; we will call this the GC+FES30 simulation. Because the tidal paths are not exactly closed, this effectively changes the initial positions of the particles in this simulation, allowing us to explore how small changes in initial position impact the final distribution. We perform this simulation to test the null hypothesis that changes in particle concentrations on time scales of years are due to different initial conditions, rather than the constant presence of the tides.

Finally, we use an artificial antibeaching boundary current that pushes particles away from the coast and thus prevents them from beaching (in our simulations, a particle beaches if it reaches a land point and gets stuck because there are no flow field data there.) This antibeaching current is normal to the coastline and has a magnitude of 1 m/s at the coast and is zero everywhere else. The main reason to implement the antibeaching current is to provide us with more particles to analyze, as comparisons of GC simulations with and without the antibeaching current show that without it, approximately 40% of the particles beach and that there are no significant differences in the results in the open ocean (see Figure S4 in the supporting information).

In all simulations, we track the particles from our initial distribution for 13 years (1 January 2002 to 1 January 2015; the full extent of the GlobCurrent data set), with time step Δt=30 minutes. Every 2 days, the position of each particle (longitude and latitude) and the distance it has traveled at that time since the beginning of the simulation are saved.

## 3 Results

### 3.1 Particle Density

**Figure 1:**
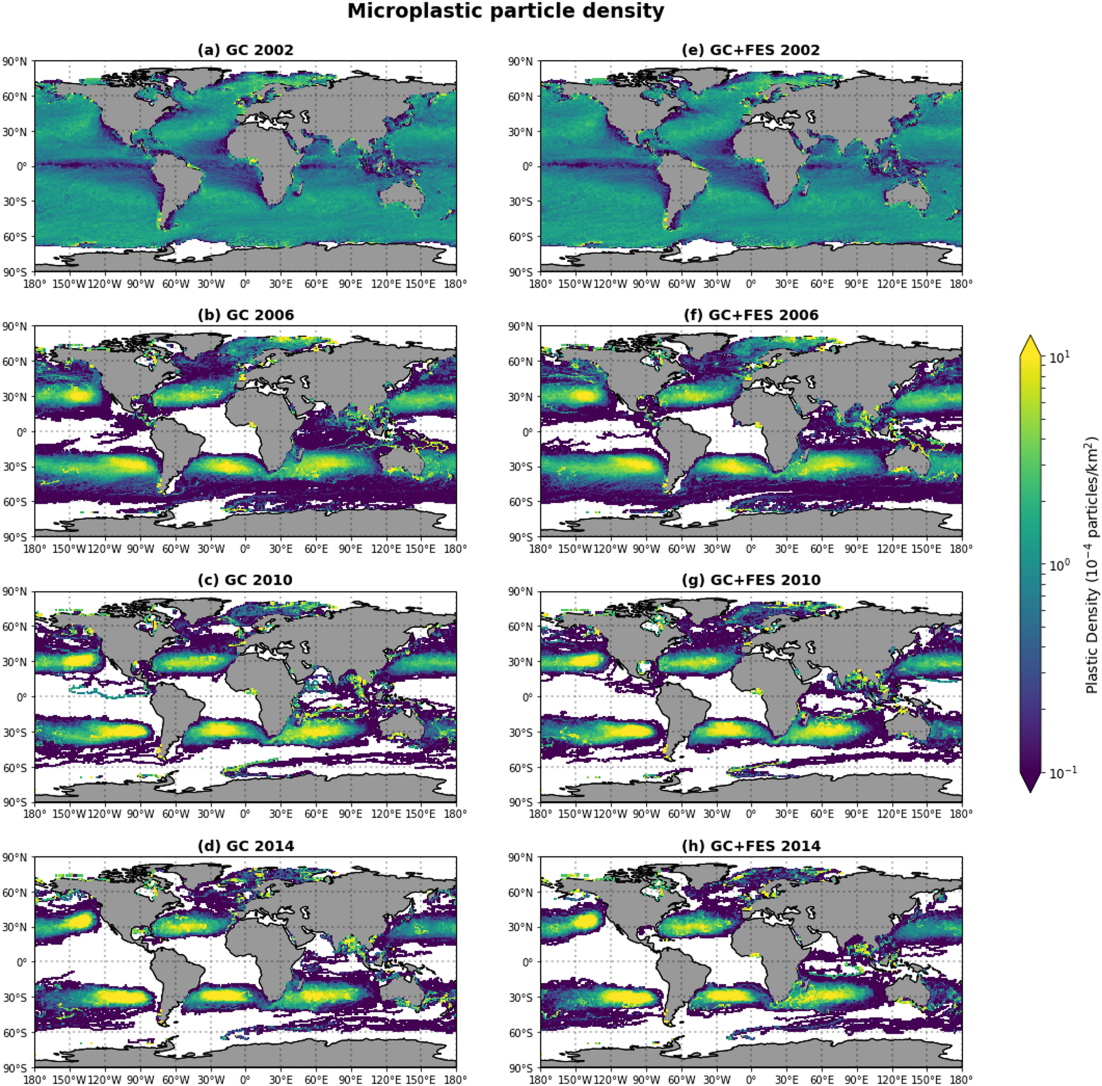
The average microplastic particle density for four different years of the simulations without barotropic tidal currents (a–d) and with the main barotropic tidal currents (e–h).

To study the fate of microplastic in our simulations, we investigate the evolution of the density of plastic in the global ocean from its initial uniform distribution, averaged over 1 year and on a global grid of 1° × 1° bins. The results for the GC simulation and the GC+FES simulation are shown for four different years of the simulations in Figure 1 (note the logarithmic scale). In the FES simulation, the density remains virtually unchanged over time from the initial density; see Figure S5 in the supporting information. The spatial patterns for the two simulations are very similar. In both situations, we see the formation of accumulation regions in the centers of all five subtropical gyres, which is in agreement with observations (e.g., Cózar et al., 2014; Lebreton et al., 2012; Maximenko et al., 2012; van Sebille et al., 2012). For the years 2010 and 2014 (the 9th and 13th years of the simulations, respectively), some differences can be observed, especially in semienclosed seas like the Gulf of Mexico and the Bay of Bengal.

**Figure 2:**
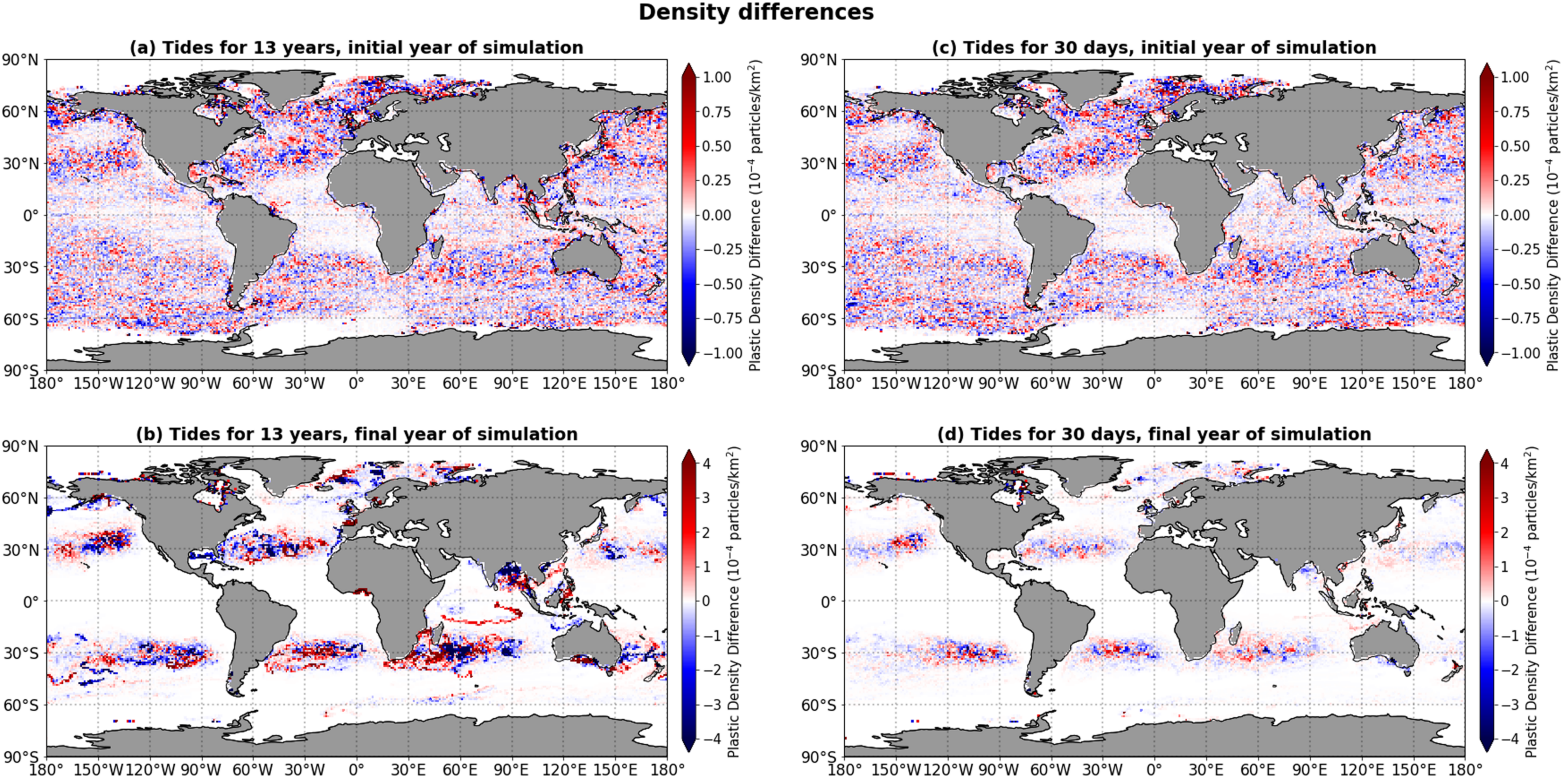
The average microplastic particle density in the simulation with the main barotropic tidal currents minus the average density in the simulation without tidal currents, for the initial and final years of the simulations. Panels (a) and (b) show the results of the GC+FES simulation (tidal currents included during the entire simulation); panels (c) and (d) show the results of the GC+FES30 simulation (tidal currents included only for the first 30 days of the 13‐year simulation).

To investigate the differences between the results of the simulations without the tides (GC), with the tides for the full simulation length (GC+FES), and with the tides for only the first 30 days (GC+FES30), we consider the density differences between these simulations. For the initial and the final years of the simulation (2002 and 2014, respectively), the year‐averaged particle density per bin in the GC+FES simulation minus the average density in the GC simulation are shown in Figures 2a and 2b (note the different scales in the two panels). The GC+FES30 density minus the GC density for the initial and final years is shown in Figures 2c and 2d. The mean absolute density differences are 1 order of magnitude smaller than the mean density itself.

Relatively large density differences are observed in the accumulation zones in the centers of the five gyres, but the pattern does not have uniform sign. However, a number of uniformly colored areas outside the large subtropical accumulation zones can be identified. The densities are lower in the GC+FES simulation than in the GC simulation in five areas: the Gulf of Mexico, the Barents Sea, the Irish Sea, the northernmost part of the Bay of Bengal, and the South China Sea along the coast of Vietnam. There are also five areas with higher densities when tides are taken into account: the Baltic Sea between Denmark and Sweden, the Bay of Biscay, a small coastal part in the Gulf of Guinea stretching from Ghana to Nigeria, the Great Australian Bight, and a rather large area to the southeast of Africa, in the region of the Agulhas Current. Although some of these areas (Irish Sea, Baltic Sea, Bay of Biscay, South China Sea, and Gulf of Guinea) are very small in terms of surface area, they are interesting because they are uniformly colored and are surrounded by regions with zero differences. Note that these hot spots are all semienclosed seas and/or near‐coastal regions, although they are not necessarily the regions with the largest tidal velocity amplitudes (see also Figures S1 and S2 in the supporting information for maps of the tidal velocity amplitudes).

To test whether the observed spatial patterns in the density differences between the GC+FES simulation and the GC simulation are caused by the presence of tidal currents, we compare the results to the density differences between the GC+FES30 simulation and the GC simulation (Figures 2c and 2d). The spatial patterns for the initial year are very similar for both cases. However, in the GC+FES30 simulation the differences in the final year are smaller in magnitude than in the GC+FES simulation, and the aforementioned uniformly blue and red hot spots in the final year (Figure 2b) are only observed in the GC+FES simulation and not in the GC+FES30 simulation (Figure 2d). This suggests that these hot spots are a result of long‐term advection by the tidal currents.

### 3.2 Total Distance Covered by Particles

In the previous section, we have seen that the effects of tidal currents on the accumulation of floating microplastic in the open ocean are very limited. While the tidal currents do move plastics around, the periodic nature of these tidal currents seems to have little net effect. To further quantify the impact of the tidal currents on plastic transport, it is insightful to investigate the net distance traveled by the particles.

**Figure 3:**
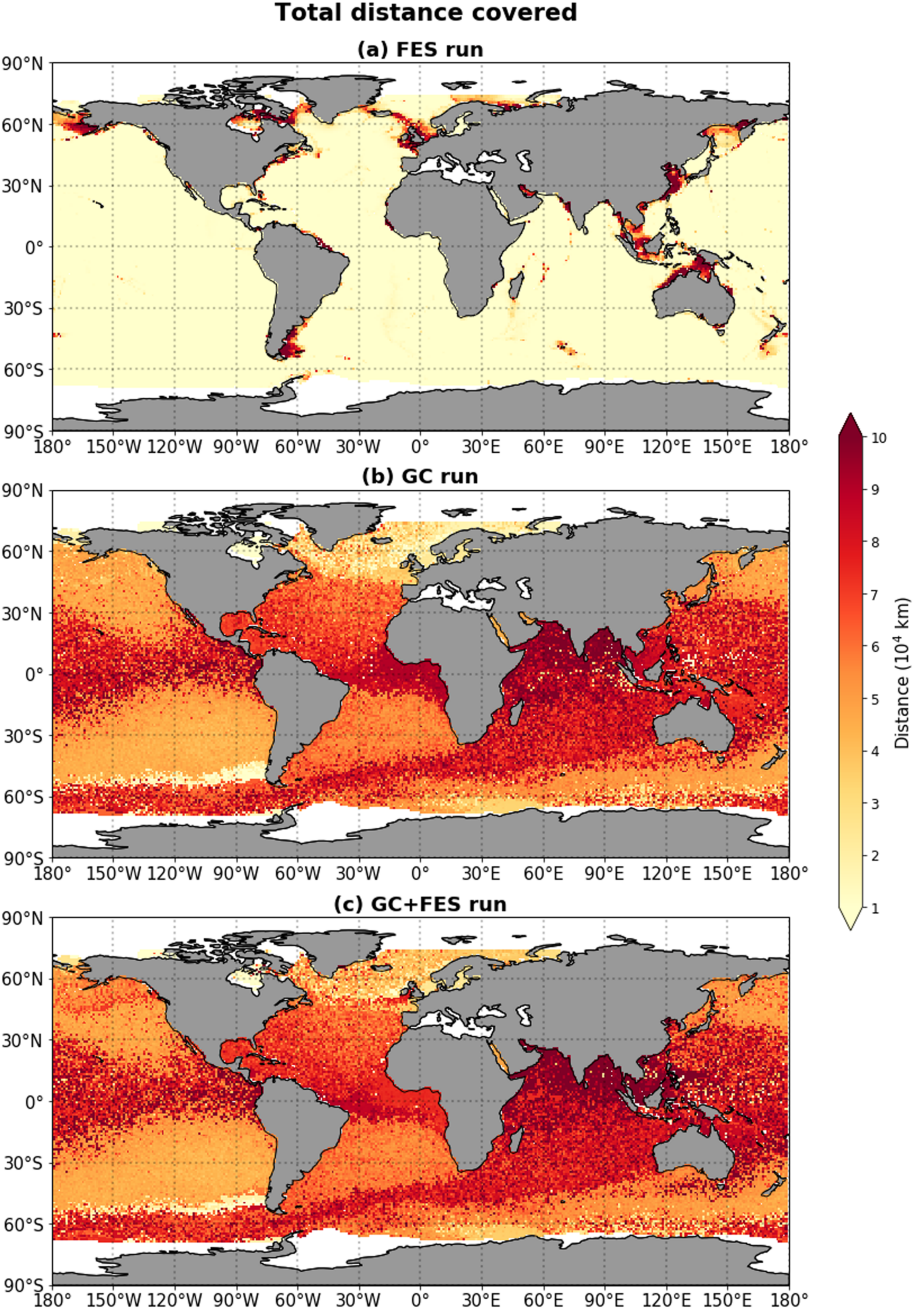
(a–c) The total distance covered by particles at the end of the simulations (i.e., after 13 years) for three different simulations, shown as a function of the particle release location.

Figure 3 shows the total distance covered at the end of the 13‐yearlong simulation as a function of the particle release location, for the three different simulations. In Figure 3a, we see that in the FES simulation, there is a clear discrepancy between coastal regions and areas in the open ocean. There are a few coastal regions where the particles that start there travel relatively far: $10^{5}$ km, as opposed to $10^{4}$ km in the open ocean (where the value of the distance traveled is also almost uniform). The regions where the particles traveled furthest in the FES simulation are precisely the coastal areas where the main tidal currents have the biggest amplitude (see also Figures S1 and S2 in the supporting information). Because of the small tidal amplitudes and the elliptic and closed nature of the tidal paths in the open ocean, particles in this FES‐only simulation do not cover much distance unless they are released in coastal areas. Figure 3b shows that the particles in the GC simulation in general cover more distance than in the FES simulation. This is as expected, since the magnitude of the geostrophic and Ekman currents is almost everywhere 1 or 2 orders of magnitude higher than the amplitudes of the tidal currents. Figure 3c, which shows the results from the GC+FES simulation, looks very similar to Figure 3b, with a few notable differences: particles released around Southeast Asia cover more distance in the GC+FES simulation than in the GC simulation, and particles released around the Equator near the Gulf of Guinea cover less distance in the GC+FES simulation than in the GC simulation. The Gulf of Guinea was recently also highlighted as a region of potentially high plastic concentrations by Mountford and Morales Maqueda (2019).

**Figure 4:**
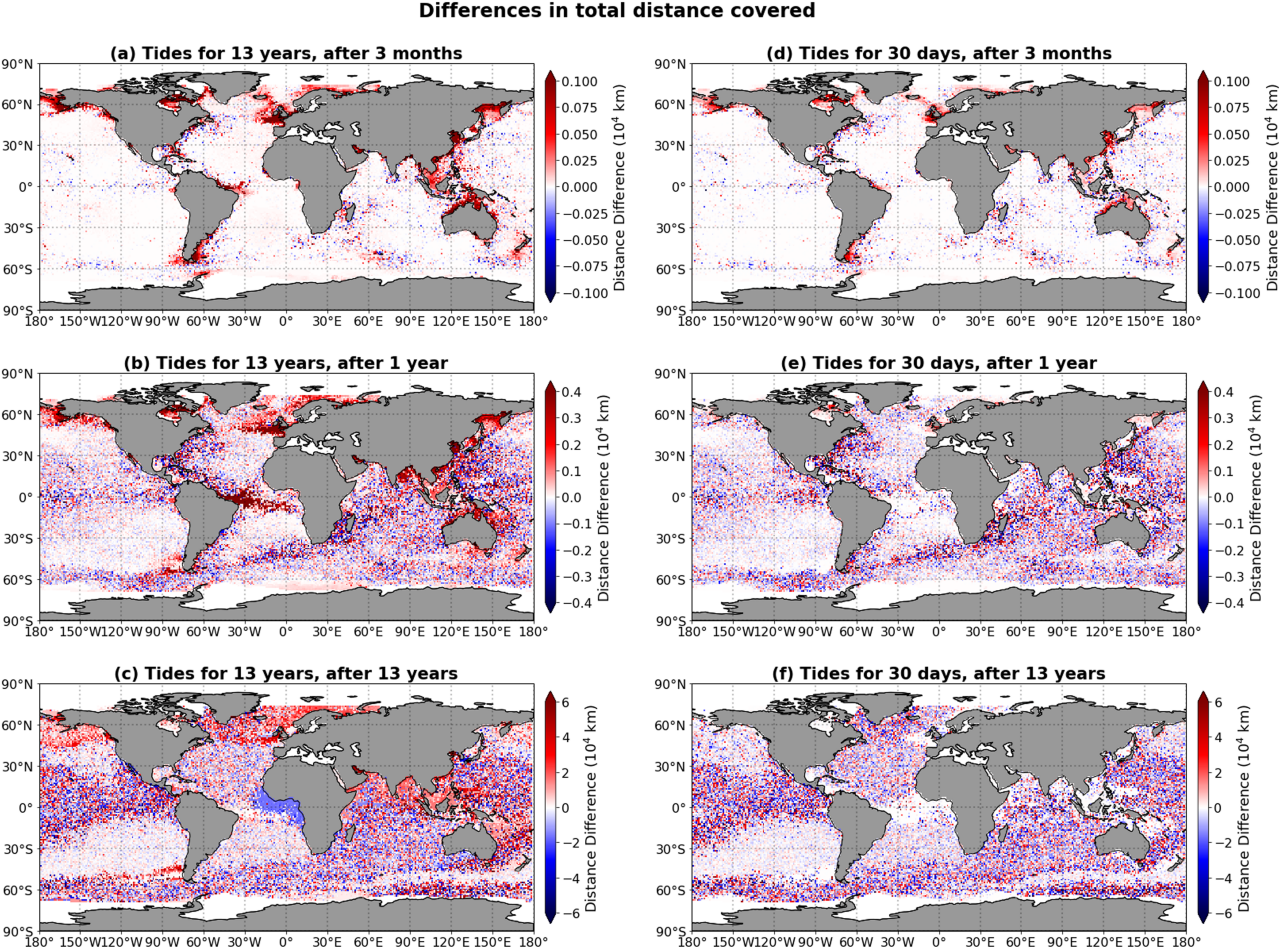
The total distance covered by particles in the simulation with the main barotropic tidal currents minus the total distance covered in the simulation without tidal currents, shown as a function of the release location, for different times after the start of the simulations. Panels (a)–(c) show the results of the GC+FES simulation (tidal currents included during the entire simulation); panels (d)–(f) show the results of the GC+FES30 simulation (tidal currents modeled only for the first 30 days of the 13‐year simulation).

The distances covered in the GC+FES simulation and the GC+FES30 simulation minus the distances covered in the GC simulation are shown in Figure 4 for different times after the start of the simulations: after 3 months, after 1 year, and after 13 years (at the end of the simulations). Note that the panels have different scales. Red indicates that a particle released at that location covers more distance in the simulation with the tides than in the simulation without these, blue that it covers less distance. After 3 months, a clear structure is visible: particles released in areas where the amplitudes of these main tidal currents are large cover more distance in the GC+FES simulation and in the GC+FES30 simulation than in the GC simulation. So on time scales of months, the tidal currents clearly influence the distance covered by the microplastic particles released in coastal regions where the tidal amplitudes are high.

After 1 year, there is still some spatial structure in the differences in distance covered in the GC+FES simulation; the regions with large differences after 3 months are still present after 1 year. In the rest of the ocean, noisy patterns form. The structures that were present after 3 months in the GC+FES30 simulation have disappeared, and the observed noisy patterns look the same as for the GC+FES simulation.

After 13 years, there is little structure left in the distance differences. The differences between the GC+FES simulation and the GC simulation show a few uniformly colored areas: the latitudinal band between 45°N and 75°N, the region along the southern and southeastern coasts of Asia, and a small region off the west coast of Chile are mostly red. In all these regions, the particles cover more distance in the GC+FES simulation than in the GC simulation. Only one uniformly blue area can be identified, namely, a rather large area along the west coast of Africa, in the Gulf of Guinea. Here particles cover less distance in the GC+FES simulation than in the GC simulation. None of these areas are observed for the GC+FES30 simulation; here, the differences appear more noisy after 13 years. It seems therefore that the uniformly red or blue hot spots form because of the main tidal currents, whereas the small‐scale noisy patterns do not: they also develop if there has only been a short period of disturbance in the beginning of a simulation with only geostrophic and Ekman currents after these initial disturbances.

**Figure 5:**
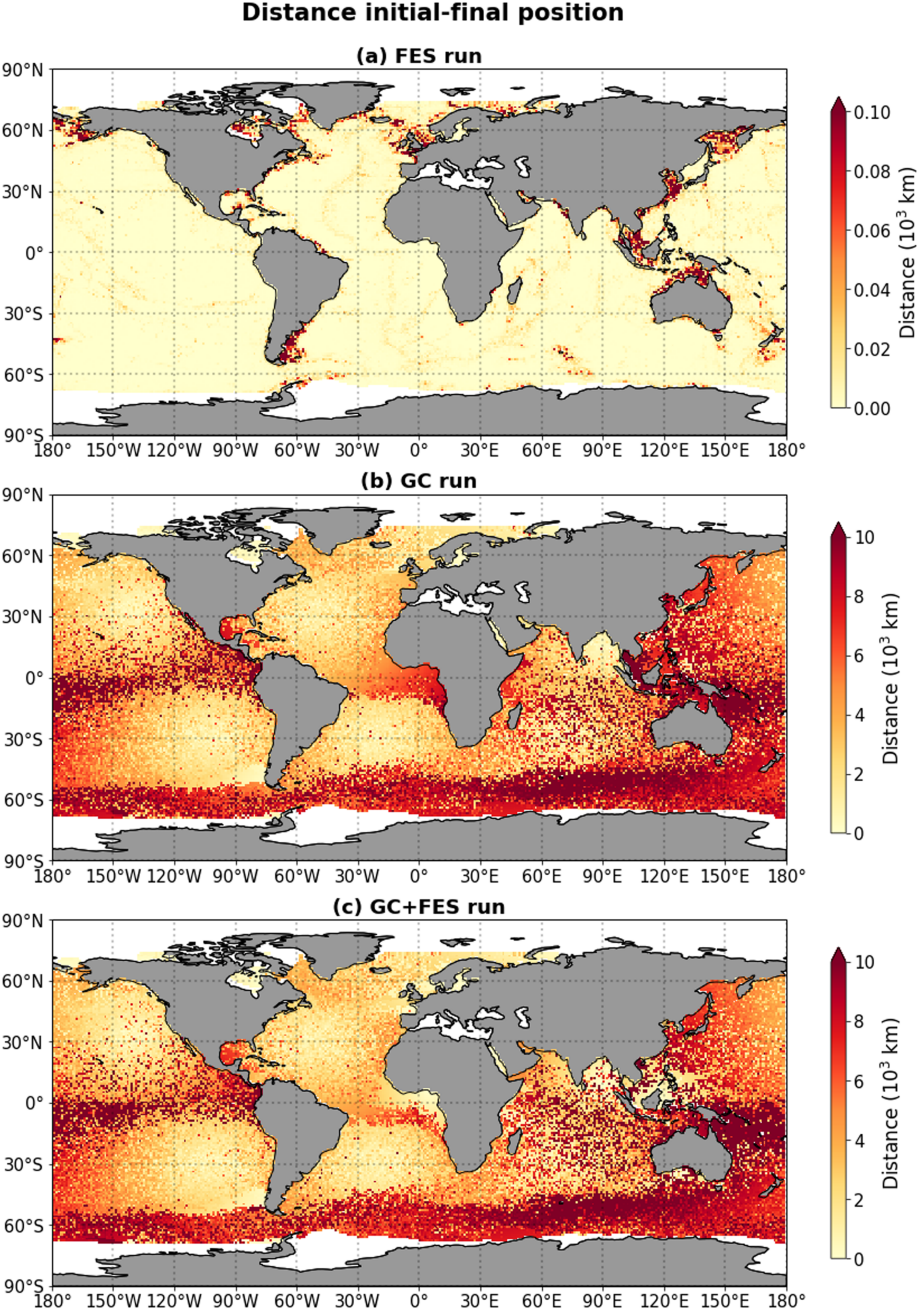
(a–c) The distance between the position of particles at the end of the simulations (i.e., after 13 years) and their initial positions, for three different simulations, shown as a function of the particle release location.

**Figure 6:**
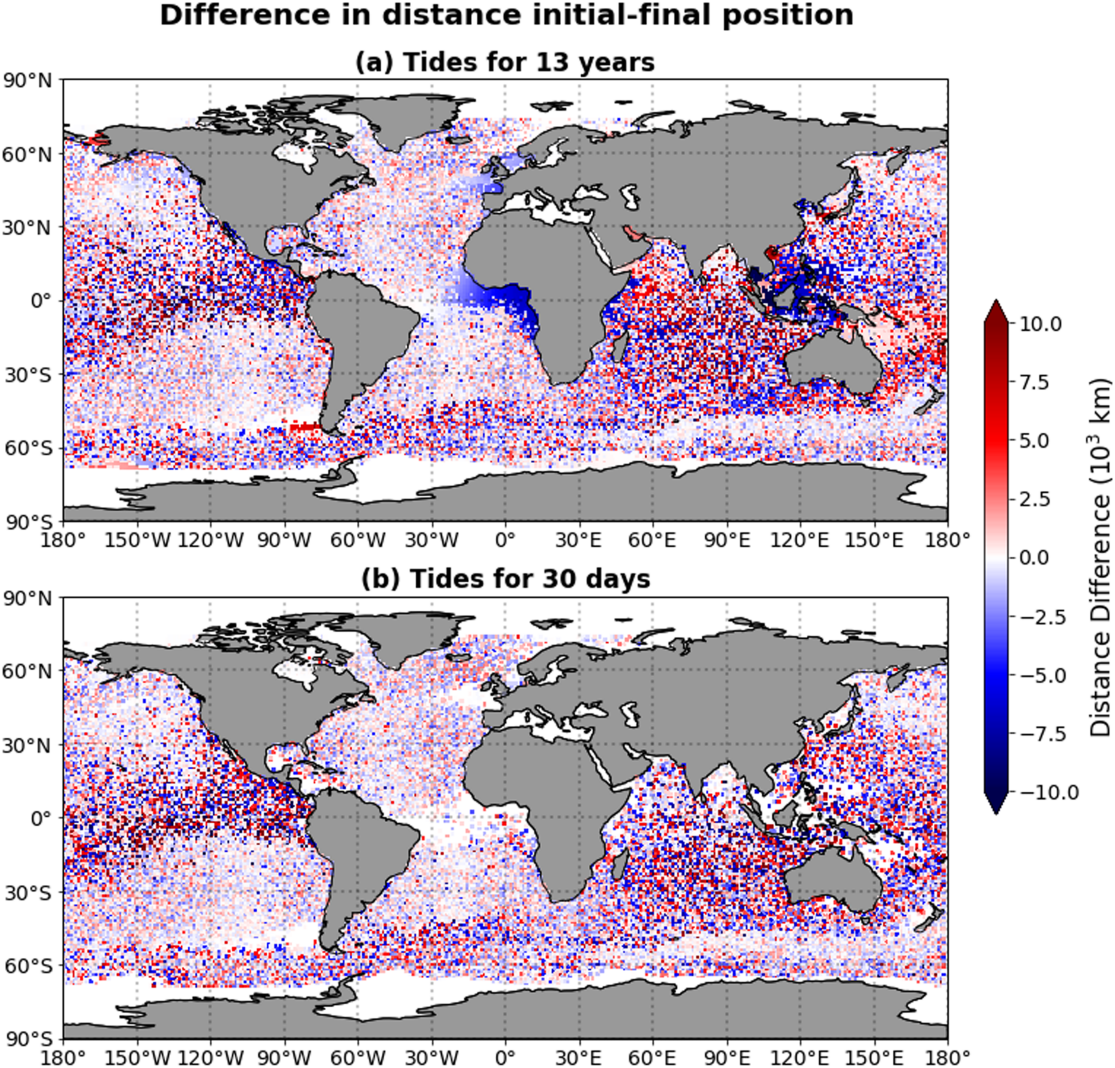
The distance between particles' final and initial positions in the simulation with the main barotropic tidal currents minus the distance between the final and initial positions in the simulation without tidal currents, shown as a function of the release location. Panel (a) shows the results of the GC+FES simulation (tidal currents included during the entire simulation); panel (b) shows the results of the GC+FES30 simulation (tidal currents modeled only for the first 30 days of the 13‐year simulation).

Since the tidal currents are periodic, they cause oscillatory motion of particles. With every oscillation, the total distance a particle covers increases, but the particle does not necessarily move far from its original location. Figure 5 shows the Haversine distance (Sinnott, 1984) between the particles' initial and final positions for the different simulations, and Figure 6 shows the difference between this distance in the runs with and without tides. Note that for the FES simulation, the distance between a particle's initial and final position depends strongly on where in the tidal cycle the start and end points are. From these figures, we can identify two interesting regions: in the Gulf of Guinea and around Southeast Asia, particles end up closer to their initial location in the GC+FES simulation than in the GC simulation. In both of these areas, there are also significant differences between the total distance covered in the GC+FES simulation and the GC simulation (see Figure 4); in the Gulf of Guinea, particles cover less distance in the GC+FES simulation than in the GC simulation, whereas around Southeast Asia they cover more distance in the GC+FES simulation than in the GC simulation.

### 3.3 Separation Between Particles

To further investigate the transport of microplastic particles advected with and without tidal currents, we analyze the separation of particle pairs in the GC simulation and the GC+FES simulation and also the separation in the GC simulation and the GC+FES30 simulation. This separation is the distance between a pair of particles that were released at the same location and time but advected with different velocity fields (e.g., Qin et al., 2014), calculated using the Haversine great‐circle distance (Sinnott, 1984).

**Figure 7:**
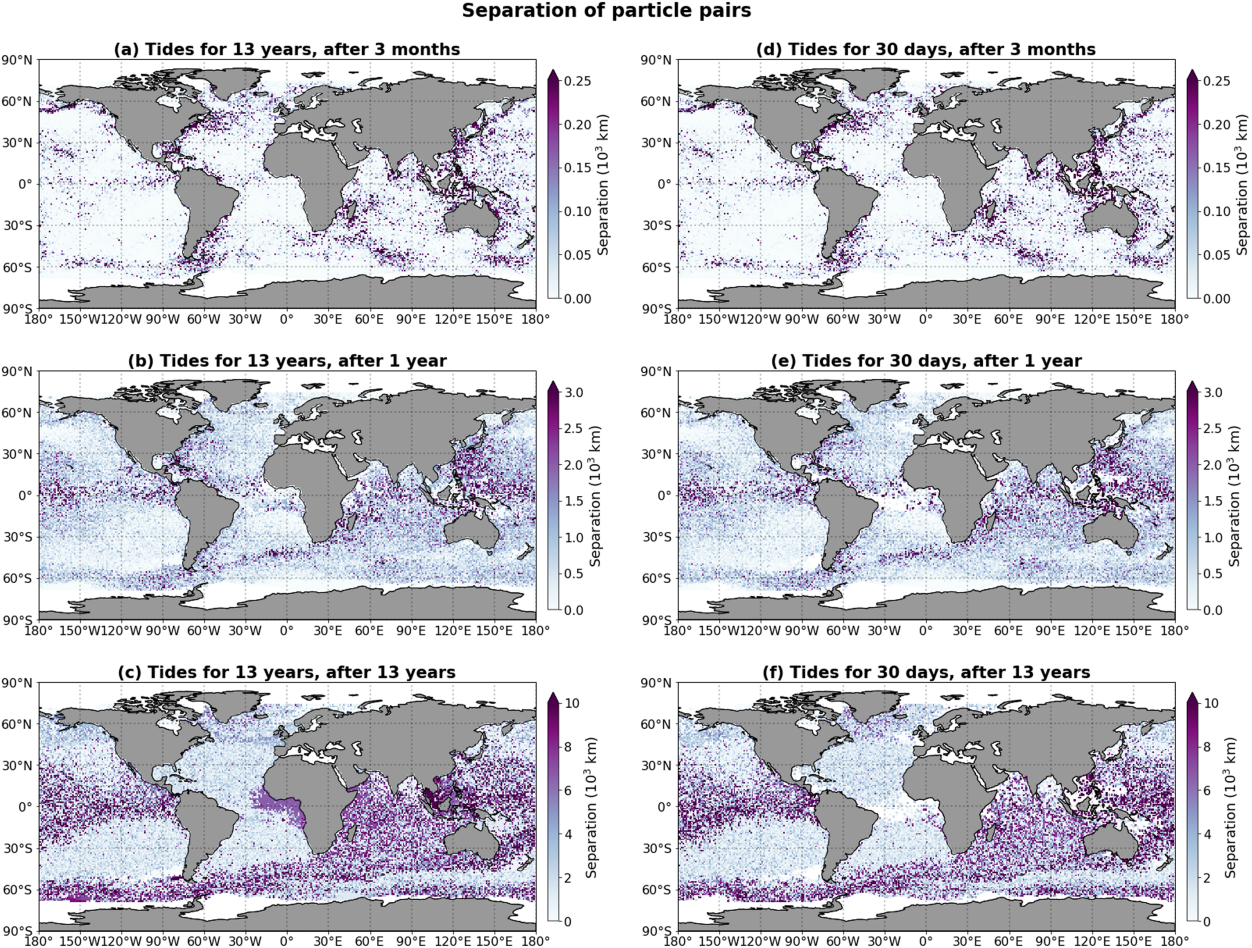
The separation between particles in the simulation with the main barotropic tidal currents and the simulation without tides, shown as a function of the release location, for different times after the start of the simulations. Panels (a)–(c) show the results of the GC+FES simulation (tidal currents included during the entire simulation); panels (d)–(f) show the results of the GC+FES30 simulation (tidal currents modeled only for the first 30 days of the 13‐year simulation).

In Figure 7, the separation is plotted as a function of release location of the particle pairs, at 3 months, 1 year, and 13 years after the beginning of the simulations. Note the different scales of the panels. After 3 months, no clear coherent structures are visible.

After 1 year and after 13 years, the patterns are similar to those observed in the differences in distance traveled (Figure 4): the areas with the largest separations largely correspond to the regions with large absolute differences in distance traveled between the two simulations. The separations are in general smaller than the differences in distance traveled. This can be explained by the periodic nature of tidal currents, as mentioned previously in section 3.2; the total distance covered by a particle always increases with time, but the distance from its original location does not necessarily do so.

The spatial structures for the GC+FES simulation and for the GC+FES30 simulation are very similar for all times (Figure 7). The only areas where differences are observable after 13 years are a region in Southeast Asia around Indonesia and the Philippines (largest separations in the Gulf of Thailand) and an area in the Atlantic near the Gulf of Guinea. In both regions, the separation is relatively large for the GC+FES simulation but is zero or at least smaller for the GC+FES30 simulation. Both of these regions were also notable regions in the results of differences in distance traveled (Figure 4).

## 4 Conclusions

Here, we have analyzed the effect of tidal currents on the transport and distributions of floating microplastic in the open ocean. We found that apart from some areas in coastal regions and semienclosed seas, these tides have virtually no effect on particle transport and distributions, especially on multiyear time scales. This “negative result” can be considered quite good news for the community performing numerical simulations of marine plastic litter (Hardesty et al., 2017), as it means that at least in the open ocean, tides do not have to be taken into account.

Note that here we focus only on the barotropic tides. In contrast, the (nonstationary) internal tides could affect dispersion of floating microplastic (van Sebille et al., 2020), especially on scales below a few hundred kilometers where they can create surface convergences (e.g., Shanks et al., 2000). However, as internal tides are very different from the barotropic tides, they are beyond the scope of this study.

Here, we took only the M ${}_{2}$, S ${}_{2}$, K ${}_{1}$, and O ${}_{1}$ currents (which together make up two thirds of the total tidal current amplitudes almost everywhere in the ocean) into account in the model of tidal currents. By doing this, we neglected the smaller tidal constituents that could influence the outcomes of the simulations. We should therefore be careful in drawing quantitative conclusions from our simulations. Nevertheless, it is reasonable to assume that our qualitative conclusions about the influence of tidal currents on transport of microplastic in the open ocean are robust and that adding more tidal constituents, which have smaller velocity amplitudes than the four constituents already included, would not alter these conclusions drastically.

A logical next step would be to further analyze the effect of tides on plastic dispersion in shelf seas and coastal regions. However, recent studies (Cancet et al., 2019; Feng et al., 2018) showed that the GlobCurrent data set agrees better with observations in the open ocean than in coastal regions; its spatial resolution is too coarse in coastal regions to yield accurate estimates of coastal currents, and its dependence on altimetry means that geostrophic flow is poorly resolved near coastlines where altimetry is less accurate. Regional models will therefore need to be used to track microplastic in coastal seas, for example, TPXO8‐atlas (https://www.tpxo.net/global/tpxo8-atlas), ideally also with tide‐current interactions.

Another point that requires more thorough investigation is the simulation of particle beaching (Hardesty et al., 2017). The artificial antibeaching current used in this study was implemented to get more robust statistics, but it does not describe a real physical process. Comparisons of simulations (with only GlobCurrent) with and without this antibeaching current show that particle densities in the open ocean show no significant differences, but without the antibeaching current, higher particle densities are found along the shores of the continents (see Figure S4 in the supporting information). This simulation without antibeaching current, however, is not a very realistic model of coastal processes either because particles simply get “stuck”. Therefore, in order to draw conclusions about the influence of tidal currents on microplastic transport and distributions in coastal regions, studies of these regions with regional models and an explicit physical model for particle beaching are needed. The particle tracking in these models could of course still be based on the code developed here for explicit computation of tidal currents from FES data.

These coastal areas of course are also very important for the fate of microplastics. The transport and residence time of microplastics in coastal regions could be considerably influenced by strong barotropic tidal currents. An example of such influence is that stranded plastic particles can easily be washed back into sea during spring tides (Zhang, 2017). Furthermore, observations of floating plastic debris in the surface waters of the Tamar Estuary (UK) reported a significant difference in the plastic particle's size frequency distribution between the spring and neap tides; during spring tides, more fragments of larger size were observed (Sadri & Thompson, 2014). While this study suggests that the location of the open‐ocean accumulation zones is relatively insensitive to tides, the amount of plastics ending up in the open ocean will depend significantly on coastal dynamics, of which tidal currents are an important component. Identifying and studying these influences of tidal currents explicitly could help us increase our understanding of the pathways and fate of microplastics in coastal environments and the amount that ends up in the open ocean.

## Supporting information



Supporting Information S1

## References

[jgrc23811-bib-0001] Andrady, A. L. (2011). Microplastics in the marine environment. Marine Pollution Bulletin, 62(8), 1596–1605. 21742351 10.1016/j.marpolbul.2011.05.030

[jgrc23811-bib-0002] Auta, H. S. , Emenike, C. U. , & Fauziah, S. H. (2017). Distribution and importance of microplastics in the marine environment: A review of the sources, fate, effects, and potential solutions. Environment International, 102, 165–176. 28284818 10.1016/j.envint.2017.02.013

[jgrc23811-bib-0003] Cancet, M. , Griffin, D. , Cahill, M. , Chapron, B. , Johannessen, J. , & Donlon, C. (2019). Evaluation of GlobCurrent surface ocean current products: A case study in Australia. Remote Sensing of Environment, 220, 71–93.

[jgrc23811-bib-0004] Carrère, L. , Lyard, F. , Cancet, M. , Guillot, A. , Picot, N. , & Dupuy, S. (2015). FES2014: A new global tidal model, Presented at the Ocean Surface Topography Science Team Meeting, Reston, USA.

[jgrc23811-bib-0005] Cole, M. , Lindeque, P. , Halsband, C. , & Galloway, T. S. (2011). Microplastics as contaminants in the marine environment: A review. Marine Pollution Bulletin, 62(12), 2588–2597. 22001295 10.1016/j.marpolbul.2011.09.025

[jgrc23811-bib-0006] Compa, M. , Alomar, C. , Wilcox, C. , van Sebille, E. , Lebreton, L. , Hardesty, B. D. , & Deudero, S. (2019). Risk assessment of plastic pollution on marine diversity in the Mediterranean Sea. Science of The Total Environment, 678, 188–196. 31075585 10.1016/j.scitotenv.2019.04.355

[jgrc23811-bib-0007] Cózar, A. , Echevarria, F. , González‐Gordillo, J. I. , Irigoien, X. , Ubeda, B. , Hernandez‐Leon, S. , Palma, A. T. , Navarro, S. , de Lomas, J. G. , Ruiz, A. , de Puelles, M. L. F. , & Duarte, C. M. (2014). Plastic debris in the open ocean. Proceedings of the National Academy of Sciences, 111(28), 10,239–10,244. 10.1073/pnas.1314705111 PMC4104848 24982135

[jgrc23811-bib-0008] Desforges, J.‐P. W. , Galbraith, M. , Dangerfield, N. , & Ross, P. S. (2014). Widespread distribution of microplastics in subsurface seawater in the NE Pacific Ocean. Marine Pollution Bulletin, 79(1), 94–99. 24398418 10.1016/j.marpolbul.2013.12.035

[jgrc23811-bib-0009] Feng, H. , Vandemark, D. , Levin, J. , & Wilkin, J. (2018). Examining the accuracy of GlobCurrent upper ocean velocity data products on the northwestern Atlantic shelf. Remote Sensing, 10(8), 1205.

[jgrc23811-bib-0010] Foreman, M. G. G. (1977). Manual for tidal heights analysis and prediction. Pacific Marine Science Report 77‐10.

[jgrc23811-bib-0011] Foreman, M. G. G. , & Henry, R. F. (1989). The harmonic analysis of tidal model time series. Advances in Water Resources, 12(3), 109–120.

[jgrc23811-bib-0012] Godin, G. (1972). The analysis of tides. Liverpool:Liverpool University Press.

[jgrc23811-bib-0013] Hardesty, B. D. , Harari, J. , Isobe, A. , Lebreton, L. , Maximenko, N. , Potemra, J. , van Sebille, E. , Vethaak, A. Dick , & Wilcox, C. (2017). Using numerical model simulations to improve the understanding of micro‐plastic distribution and pathways in the marine environment. Frontiers in Marine Science, 4(30), 1–9.

[jgrc23811-bib-0014] Iwasaki, S. , Isobe, A. , Kako, S. , Uchida, K. , & Tokai, T. (2017). Fate of microplastics and mesoplastics carried by surface currents and wind waves: A numerical model approach in the Sea of Japan. Marine Pollution Bulletin, 121(1), 85–96. 28559056 10.1016/j.marpolbul.2017.05.057

[jgrc23811-bib-0015] Jalón‐Rojas, I. , Wang, X. H. , & Fredj, E. (2019). A 3D numerical model to Track Marine Plastic Debris (TrackMPD): Sensitivity of microplastic trajectories and fates to particle dynamical properties and physical processes. Marine Pollution Bulletin, 141, 256–272. 30955734 10.1016/j.marpolbul.2019.02.052

[jgrc23811-bib-0016] Lacerda, A. L. d. F. , Rodrigues, L. d. S. , van Sebille, E. , Rodrigues, F. L. , Ribeiro, L. , Secchi, E. R. , Kessler, F. , & Proietti, M. C. (2019). Plastics in sea surface waters around the Antarctic Peninsula. Scientific Reports, 9(1), 3977. 30850657 10.1038/s41598-019-40311-4 PMC6408452

[jgrc23811-bib-0017] Lange, M. , & van Sebille, E. (2017). Parcels v0.9: Prototyping a Lagrangian ocean analysis framework for the petascale age. Geoscientific Model Development, 10, 4175–4186.

[jgrc23811-bib-0018] Law, K. L. (2016). Plastics in the marine environment. Annual Review of Marine Science, 9(1), 205–229. 10.1146/annurev-marine-010816-060409 27620829

[jgrc23811-bib-0019] Lebreton, L. C.‐M. , Greer, S. D. , & Borrero, J. C. (2012). Numerical modelling of floating debris in the world's oceans. Marine Pollution Bulletin, 64, 653–661. 22264500 10.1016/j.marpolbul.2011.10.027

[jgrc23811-bib-0020] Lebreton, L. C. M. , van der Zwet, J. , Damsteeg, J.‐W. , Slat, B. , Andrady, A. , & Reisser, J. (2017). River plastic emissions to the world's oceans. Nature Communications, 8, 15611. 10.1038/ncomms15611 PMC5467230 28589961

[jgrc23811-bib-0021] Lumpkin, R. , Ozgokmen, T. M. , & Centurioni, L. (2017). Advances in the application of surface drifters. Annual Review of Marine Science, 9(1), 59–81. 10.1146/annurev-marine-010816-060641 27575739

[jgrc23811-bib-0022] Maximenko, N. , Corradi, P. , Law, K. L. , Van Sebille, E. , Garaba, S. P. , Lampitt, R. S. , Galgani, F. , Martinez‐Vicente, V. , Goddijn‐Murphy, L. , Veiga, J. M. , Thompson, R. C. , Maes, C. , Moller, D. , Lscher, C. R. , Addamo, A. M. , Lamson, M. , Centurioni, L. R. , Posth, N. , Lumpkin, R. , Vinci, M. , Martins, A. M. , Pieper, C. D. , Isobe, A. , Hanke, G. , Edwards, M. , Chubarenko, I. P. , Rodriguez, E. , Aliani, S. , Arias, M. , Asner, G. P. , Brosich, A. , Carlton, J. T. , Chao, Y. , Cook, A.‐M. , Cundy, A. , Galloway, T. S. , Giorgetti, A. , Goni, G. J. , Guichoux, Y. , Hardesty, B. D. , Holdsworth, N. , Lebreton, L. , Leslie, H. A. , Macadam‐Somer, I. , Mace, T. , Manuel, M. , Marsh, R. , Martinez, E. , Mayor, D. , Le Moigne, M. , Molina Jack, M. E. , Mowlem, M. C. , Obbard, R. W. , Pabortsava, K. , Robberson, B. , Rotaru, A.‐E. , Spedicato, M. T. , Thiel, M. , Turra, A. , & Wilcox, C. (2019). Towards the integrated marine debris observing system. Frontiers in Marine Science, 6, 447.

[jgrc23811-bib-0023] Maximenko, N. , Hafner, J. , & Niiler, P. (2012). Pathways of marine debris derived from trajectories of Lagrangian drifters. Marine Pollution Bulletin, 65, 51–62. 21696778 10.1016/j.marpolbul.2011.04.016

[jgrc23811-bib-0024] Mountford, A. S. , & Morales Maqueda, M. A. (2019). Eulerian modeling of the three‐dimensional distribution of seven popular microplastic types in the global ocean. Journal of Geophysical Research: Oceans, 124 10.1029/2019JC015050

[jgrc23811-bib-0025] Onink, V. , Wichmann, D. , Delandmeter, P. , & van Sebille, E. (2019). The role of Ekman currents, geostrophy, and Stokes drift in the accumulation of floating microplastic. Journal of Geophysical Research: Oceans, 124, 1474–1490. 10.1029/2018JC014547 31218155 PMC6559306

[jgrc23811-bib-0026] Qin, X. , van Sebille, E. , & Sen Gupta, A. (2014). Quantification of errors induced by temporal resolution on Lagrangian particles in an eddy‐resolving model. Ocean Modelling, 76, 20–30.

[jgrc23811-bib-0027] Rio, M.‐H. , Johannessen, J. , & Donlan, C. (2015). Product data handbook: The combined geostrophy + Ekman currents.

[jgrc23811-bib-0028] Rio, M.‐H. , Mulet, S. , & Picot, N. (2014). Beyond GOCE for the ocean circulation estimate: Synergetic use of altimetry, gravimetry, and in situ data provides new insight into geostrophic and Ekman currents. Geophysical Research Letters, 41, 8918–8925. 10.1002/2014GL061773

[jgrc23811-bib-0029] Sadri, S. S. , & Thompson, R. C. (2014). On the quantity and composition of floating plastic debris entering and leaving the Tamar Estuary, Southwest England. Marine Pollution Bulletin, 81(1), 55–60. 24613232 10.1016/j.marpolbul.2014.02.020

[jgrc23811-bib-0030] Schureman, P. (1958). Manual of harmonic analysis and prediction of tides. Washington:U.S. Department of Commerce, Coast and Geodetic Survey. Special Publication No. 98.

[jgrc23811-bib-0031] Shanks, A. L. , Largier, J. , Brink, L. , Brubaker, J. , & Hooff, R. (2000). Demonstration of the onshore transport of larval invertebrates by the shoreward movement of an upwelling front. Limnology and Oceanography, 45(1), 230–236.

[jgrc23811-bib-0032] Sinnott, R. W. (1984). Virtues of the Haversine. Sky and Telescope, 68(2), 159.

[jgrc23811-bib-0033] Torres, H. S. , Klein, P. , Menemenlis, D. , Qiu, B. , Su, Z. , Wang, J. , Chen, S. , & Fu, L.‐L. (2018). Partitioning ocean motions into balanced motions and internal gravity waves: A modeling study in anticipation of future space missions. Journal of Geophysical Research: Oceans, 123, 8084–8105. 10.1029/2018JC014438

[jgrc23811-bib-0034] van Sebille, E. , England, M. H. , & Froyland, G. (2012). Origin, dynamics and evolution of ocean garbage patches from observed surface drifters. Environmental Research Letters, 7(4), 1–6.

[jgrc23811-bib-0035] van Sebille, E. , Aliani, S. , Law, K. L. , Maximenko, N. , Alsina, J. M. , Bagaev, A. , Bergmann, M. , Chapron, B. , Chubarenko, I. , Cózar, A. , Delandmeter, P. , Egger, M. , Fox‐Kemper, B. , Garaba, S. P. , Goddijn‐Murphy, L. , Hardesty, B. D. Hoffman, M. J. , Isobe, A. , Jongedijk, C. E. , Kaandorp, M. L. A. , Khatmullina, L. , Koelmans, A. A. , Kukulka, T. , Laufkötter, C. , Lebreton, L. , Lobelle, D. , Martinez‐Vicente, V. , Morales Maqueda, M. A. , Poulain‐Zarcos, M. , Rodríguez, E. , Ryan, P. G. , Shanks, A. L. , Shim, W. J. , Suaria, G. , Thiel, M. , van den Bremer, T. S. , & Wichmann, D. (2020). The physical oceanography of the transport of floating marine debris. Environmental Research Reviews. 10.1088/1748-9326/ab6d7d

[jgrc23811-bib-0036] van Sebille, E. , Griffies, S. M. , Abernathey, R. , Adams, T. P. , Berloff, P. S. , Biastoch, A. , Blanke, B. , Chassignet, E. P. , Cheng, Y. , Cotter, C. J. , Deleersnijder, E. , Ds, K. , Drake, H. F. , Drijfhout, S. S. , Gary, S. F. , Heemink, A. W. , Kjellsson, J. , Koszalka, I. M. , Lange, M. , Lique, C. , MacGilchrist, G. A. , Marsh, R. , Adame, C. G. M. , McAdam, R. , Nencioli, F. , Paris, C. B. , Piggott, M. D. , Polton, J. A. , Rhs, S. , Shah, S. H. A. M. , Thomas, M. D. , Wang, J. , Wolfram, P. J. , Zanna, L. , & Zika, J. D. (2018). Lagrangian ocean analysis: Fundamentals and practices. Ocean Modelling, 121, 49–75.

[jgrc23811-bib-0037] van Sebille, E. , Wilcox, C. , Lebreton, L. , Maximenko, N. , Hardesty, B. D. , van Franeker, J. A. , Eriksen, M. , Siegel, D. , Galgani, F. , & Law, K. L. (2015). A global inventory of small floating plastic debris. Environmental Research Letters, 10(12), 1–11.

[jgrc23811-bib-0038] Wichmann, D. , Delandmeter, P. , & van Sebille, E. (2019). Influence of near‐surface currents on the global dispersal of marine microplastic. Journal of Geophysical Research: Oceans, 124, 6086–6096. 10.1029/2019JC015328 PMC6559306 31218155

[jgrc23811-bib-0039] Zhang, H. (2017). Transport of microplastics in coastal seas. Estuarine, Coastal and Shelf Science, 199, 74–86.

