# Supplementary material for: Influence of Barotropic Tidal Currents on Transport and Accumulation of Floating Microplastics in the Global Open Ocean
Source: J Geophys Res Oceans. 2020 Jan 29;125(2):e2019JC015583. doi: 10.1029/2019JC015583 (PMC7375081; doi:10.1029/2019JC015583)
Supplement: Supplementary file 1 — Supporting Information S1 [file JGRC-125-e2019JC015583-s001.pdf]

Supporting Information for "Influence of barotropic tidal currents on transport and accumulation of floating microplastics in the global open ocean"

Miriam F. Sterl[1], Philippe Delandmeter[1], and Erik van Sebille[1]

[1]Institute for Marine and Atmospheric Research, Utrecht University, Princetonplein 5, 3584 CC Utrecht, the Netherlands

Contents of this file

1. Text S1. Calculation of the main tidal currents in Parcels

2. Figures S1 to S5

Text S1. Calculation of the main tidal currents in Parcels

There exist a great number of tidal constituents; Doodson (1921) distinguished as many as 388 different constituents (Casotto & Biscani, 2004). In the Kernel developed for Parcels at `https://github.com/OceanParcels/Tides_GlobalOceanPlastic`, we compute the tidal currents using harmonic analysis of the four largest tidal constituents, for any location and time. The strongest tidal constituent is the M_2 tide, or semi-diurnal lunar tide. It is caused by forcing due to the Moon and its angular frequency is $\omega_{M_2} = 28.9841042°$/hour, which corresponds to a period of half a lunar day (12 h 25 m). Likewise, the S_2 tide, or semi-diurnal solar tide, is the result of forcing by the Sun; it has angular frequency $\omega_{S_2} = 30.0000000°$/hour, and period half a solar day (12 h) (Schureman, 1958).[1] Two other important constituents are the K_1 tide, or diurnal luni-

solar tide, and the O_1 tide, or diurnal lunar tide. These tides are related to the inclinations of the Earth's axis and the Moon's orbit. Their frequencies are $\omega_{K_1} = 15.0410686°/\text{hour}$ and $\omega_{O_1} = 13.9430356°/\text{hour}$, respectively (Schureman, 1958). Since the Moon causes the strongest tidal force,the M_2 constituent has the largest amplitude of all tidal constituents. The K_1 tide has the second-largest amplitude, which is 58.4% of the M_2 amplitude. The S_2 and O_1 tide follow in third and fourth place, respectively, with 46.6% and 41.5% of the M_2 amplitude.

Because these are the main tidal constituents, as a first approximation only the M_2, S_2, K_1 and O_1 constituents are taken into account here for the computation of tidal velocity fields. Data for the amplitudes and phase shifts of the main tidal currents are obtained from the FES2014 data set. The astronomical argument correction and nodal modulation amplitude and phase corrections can be calculated from a number of astronomical variables (Foreman, 1977):

$T(t)$, solar angle relative to Greenwich;

$h(t)$, longitude of the Sun;

$s(t)$, longitude of the Moon;

$N(t)$, longitude of the Moon's ascending node.

For our calculations, we take the origin of time t_0 to be January 1, 1900, 00:00:00 UTC. For each time t, let $\tau = t - t_0$ be the time that has passed since t_0, expressed in number of Julian centuries (36,525 days). Then the astronomical variables defined above can be

calculated as

$$T(t) = 180.0^\circ + 36525 \cdot 360.0^\circ \tau,$$

$$h(t) = 280.1895015^\circ + 36000.76892^\circ \tau,$$

$$s(t) = 277.0256206^\circ + 481267.892^\circ \tau,$$

$$N(t) = 259.1560563^\circ - 1934.1423972^\circ \tau.$$

These constants are taken from the code accompanying the FES2014 data set. They can also be found in (Doodson, 1921), where some of the values are slightly different from those in the FES2014 code (differences occurring in the third decimal); since the FES2014 code is more recent, it is assumed that the values used there are the results of more precise measurements, so these values are used for the calculations.

For the calculation of the nodal modulations, we also need four more variables I, ξ, ν and ν', which can be calculated from $N(t)$ using the following relations (Schureman, 1958):

$$\cos I = 0.91370 - 0.03569 \cos N, \quad (1)$$

$$\tan\left(\frac{N - \xi + \nu}{2}\right) = 1.01883 \tan\left(N/2\right), \quad (2)$$

$$\tan\left(\frac{N - \xi - \nu}{2}\right) = 0.64412 \tan\left(N/2\right), \quad (3)$$

$$\tan \nu' = \frac{\sin(2I)\sin(\nu)}{\sin(2I)\cos(\nu) + 0.3347}. \quad (4)$$

From these numbers, we can calculate the values of $V(t)$, $u(t)$ and $f(t)$ for each of the main tidal constituents. The formulas for these calculations are listed in Table S1.

All these formulas are taken from (Schureman, 1958). For further background and discussion on these formulas, we refer to (Godin, 1972) and (Schureman, 1958). The total eastward and northward currents due to the four main tidal constituents can now be

calculated for every location and every time, using observational data for the tidal current amplitudes and phase shifts from the FES2014 data set, and with V, u and f calculated as in Table S1.

References

Casotto, S., & Biscani, F. (2004). A fully analytical approach to the harmonic development of the tide-generating potential accounting for precession, nutation, and perturbations due to figure and planetary terms. *Bulletin of the American Astronomical Society*, *36*, 862.

Doodson, A. T. (1921). The harmonic development of the tide-generating potential. *Proceedings of the Royal Society, Series A*, *100*(704), 305–329. doi: 10.1098/rspa.1921.0088

Foreman, M. G. G. (1977). Manual for tidal heights analysis and prediction [Computer software manual]. Pacific Marine Science Report 77-10.

Godin, G. (1972). *The analysis of tides.* Liverpool: Liverpool University Press.

Schureman, P. (1958). *Manual of harmonic analysis and prediction of tides.* Washington: U.S. Department of Commerce, Coast and Geodetic Survey. (Special Publication No. 98)

Notes

1. As the M_2 and S_2 tides move in and out of phase, they cause the spring-neap cycle, a 14.8 day cycle during which the tidal forces caused by the Sun and the Moon either reinforce each other (spring tides and spring currents) or partially cancel each other (neap tides and neap currents) (Godin, 1972).

	V	u	f
$\mathbf{M}_2$	$2T - 2s + 2h$	$2\xi - 2\nu$	$1.0924 \cos^4(I/2)$
$\mathbf{S}_2$	$2T$	0	1
$\mathbf{K}_1$	$T + h - 90°$	$-\nu'$	$\sqrt{0.8965 \sin^2(I) + 0.6001 \sin(2I) \cos(\nu) + 0.1006}$
$\mathbf{O}_1$	$T - 2s + h + 90°$	$2\xi - \nu$	$2.6316 \sin(I) \cos^2(I/2)$

Table S1. The expressions for the astronomical argument correction $V(t)$, the nodal modulation phase correction $u(t)$ and the nodal modulation amplitude correction $f(t)$ for the M_2, S_2, K_1 and O_1 tidal constituents.

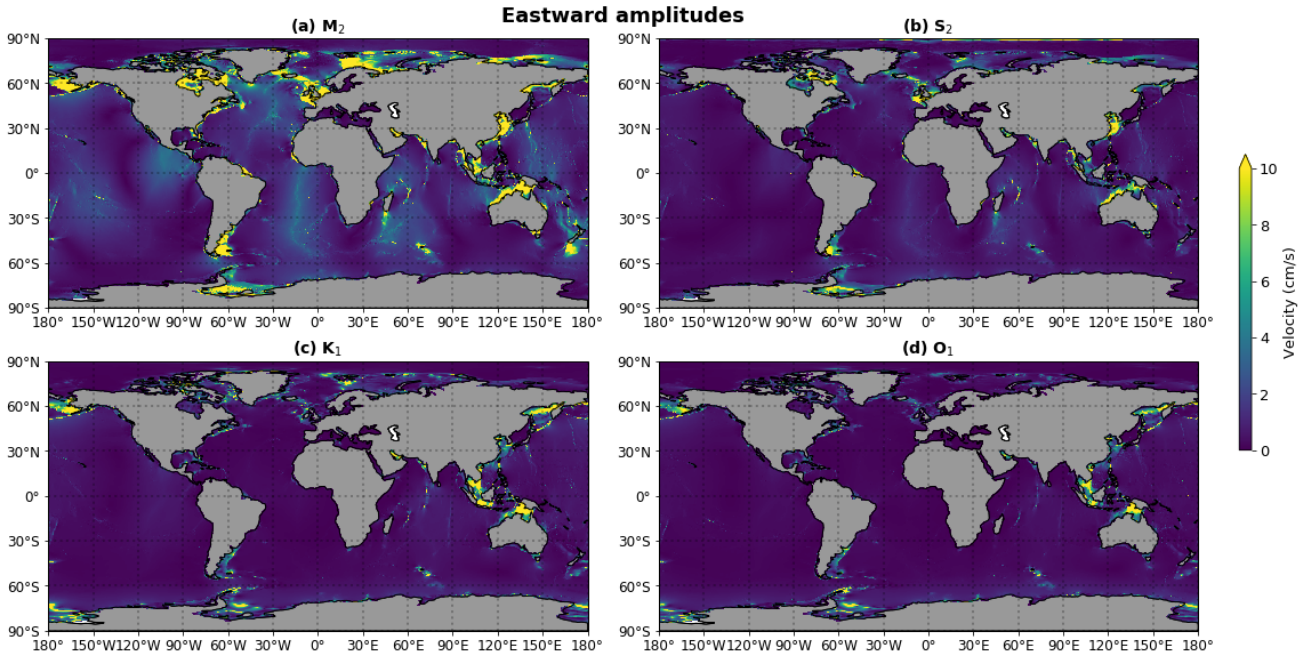

Figure S1. The eastward velocity amplitudes of the M_2, S_2, K_1 and O_1 tidal currents from the FES2014 data set.

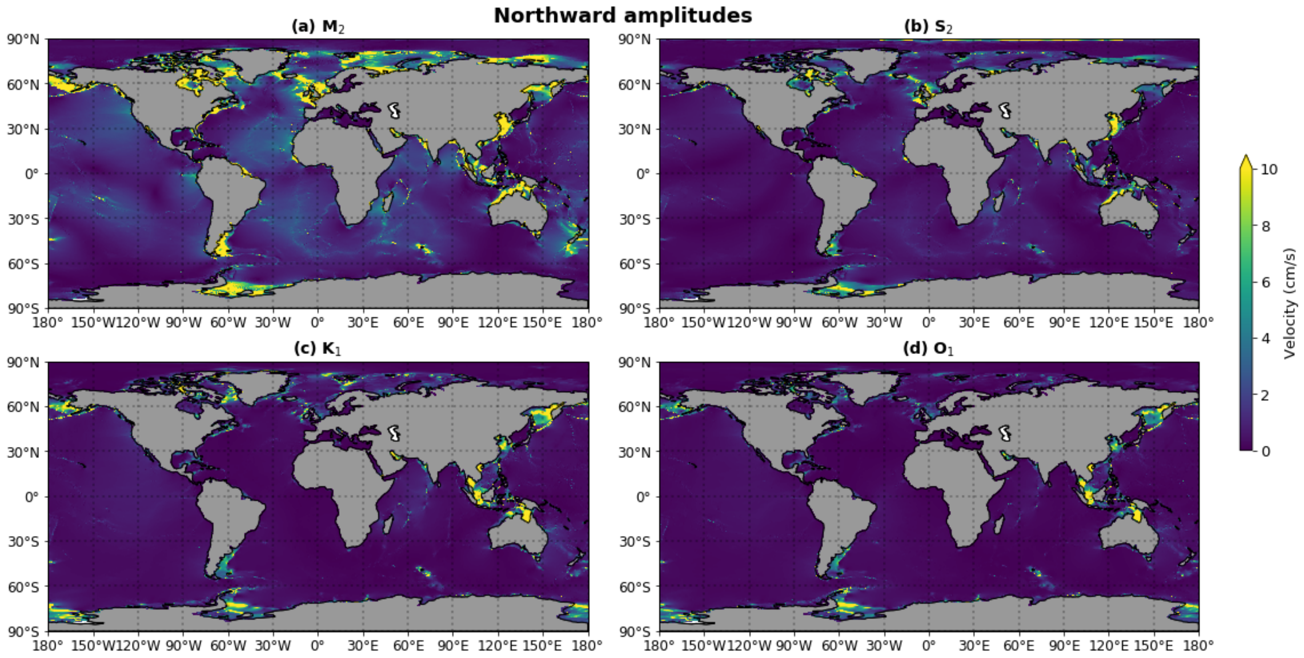

Figure S2. The northward velocity amplitudes of the M_2, S_2, K_1 and O_1 tidal currents from the FES2014 data set.

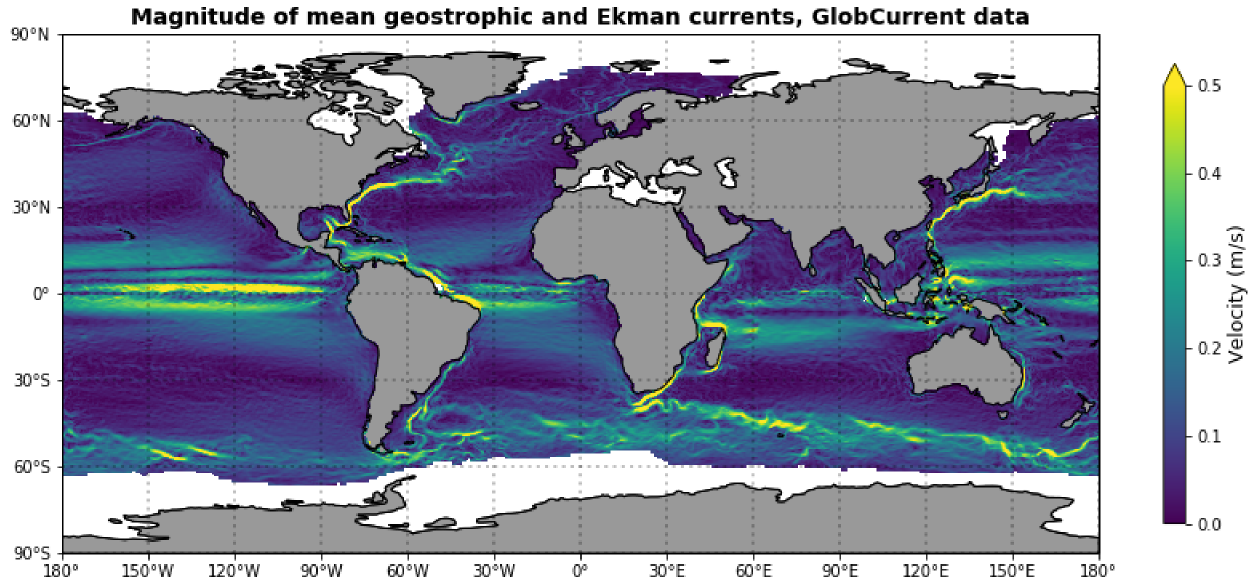

Figure S3. The magnitude of the time-averaged total surface currents (geostrophic and Ekman currents) from the GlobCurrent v3 data set. Time averages are computed for the years 2002-2014.

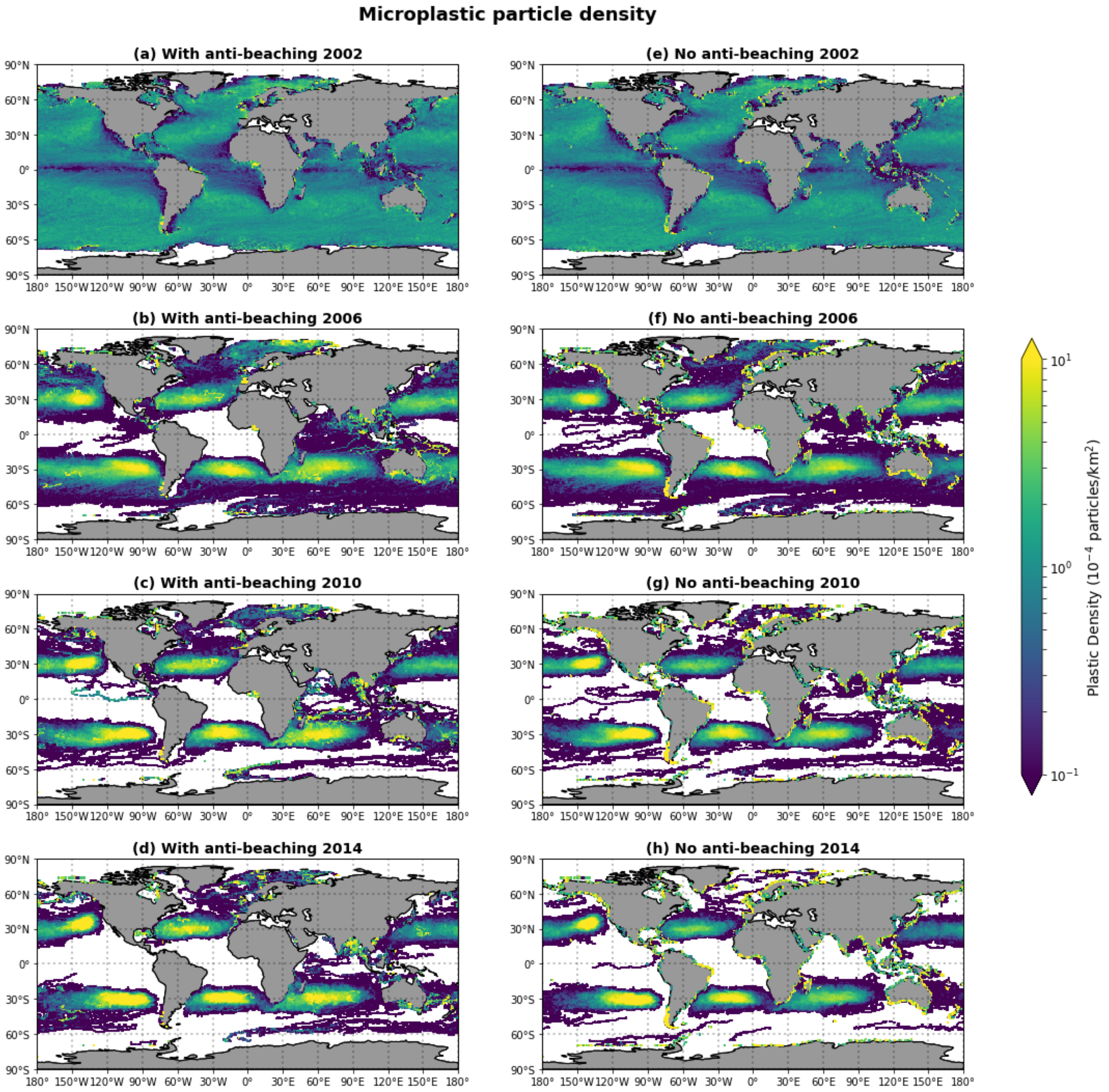

Figure S4. The average microplastic particle density for four different years of the GC simulations with artificial anti-beaching current (left column) and without the anti-beaching current (right column). This anti-beaching current is normal to the coastline and has a magnitude of 1 ms^{-1} at the coast, and is zero everywhere else.

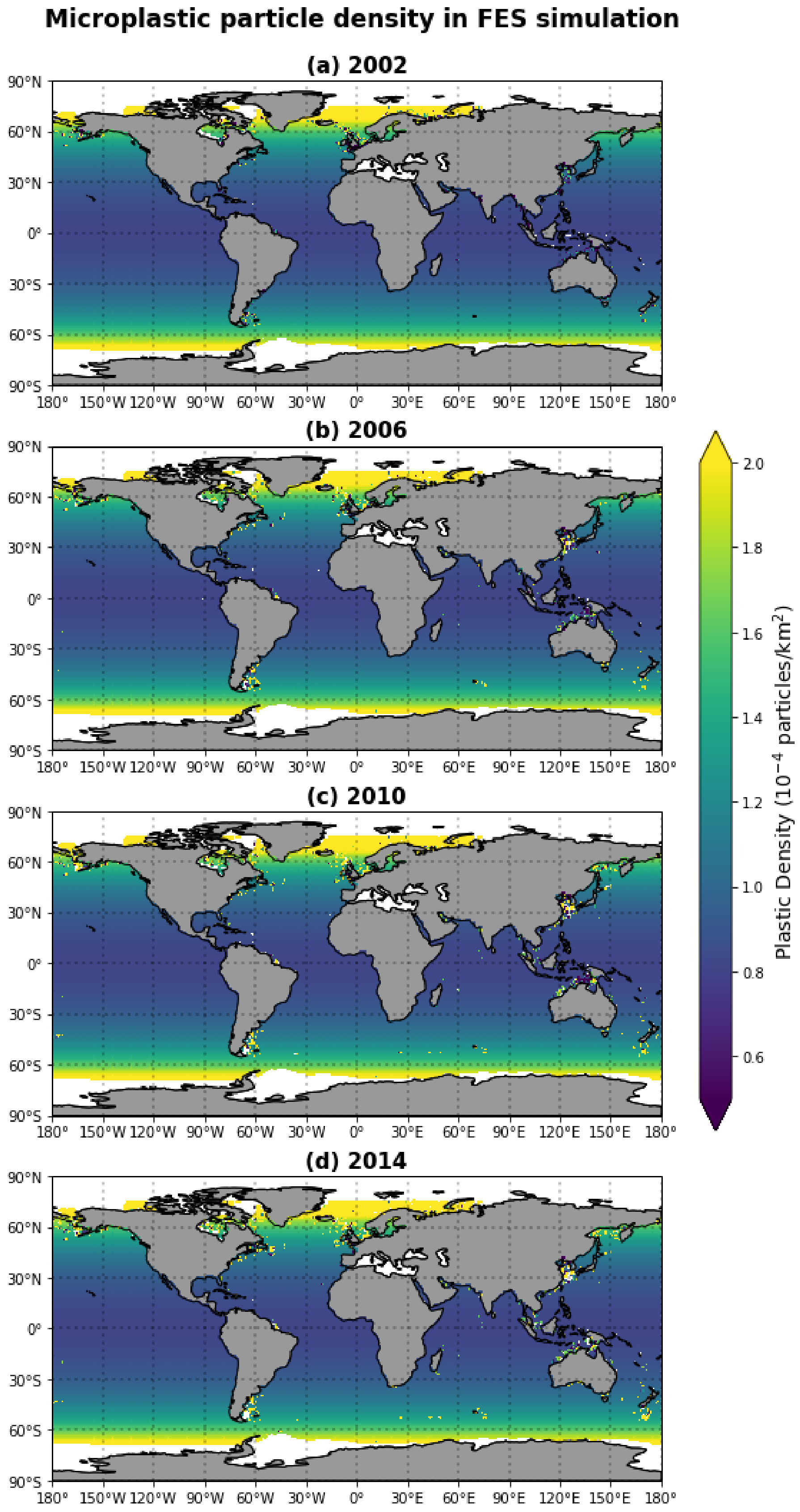

Figure S5. The average microplastic particle density for four different years of the simulation with only barotropic tidal currents.
